# Village Health and Sanitation Committees: evaluation of a capacity-building intervention in Bagalkot and Koppal districts of Northern Karnataka

**DOI:** 10.1186/1753-6561-6-S5-O24

**Published:** 2012-09-28

**Authors:** HL Mohan, Dattatreya Jere, Manjunath Dodawad, Nagaraj Ramaiah, Raghavendra Kamati, Suresh Garagatti, Ajay Gaikwad, Arin Kar, Shalini Navale, Mallika Biddappa, BM Ramesh

**Affiliations:** 1 Karnataka Health Promotion Trust, Bangalore, India; 2 Eastern Virginia Medical School, Norfolk, VA 23507, USA

## Introduction

Engaging the community in planning and monitoring of health service delivery is central to enhancing the availability, accessibility, quality, and use of the public health system. The National Rural Health Mission (NRHM) has positioned community ownership as central to its strategy, primarily through the Village Health and Sanitation Committee (VHSC). The VHSCs are village-level bodies comprised of key stakeholders in a village and serve as a forum for village planning and monitoring. VHSCs were formed (1) to ensure that no section of the village community is excluded from services, (2) to prepare a village health plan to suit local realities and necessities, (3) to provide monitoring and oversight to all village health activities; and (4) to ensure that untied funds are appropriately used for improving maternal and neonatal health in the village. There have been few efforts in the health sector that effectively evaluated the relevance and need of community participation to improve health at the village level. The Karnataka Health Promotion Trust (KHPT) carried out a 2-year capacity-building project to strengthen VHSCs in Bagalkot and Koppal districts during 2009-2011 and attempted an evaluation of project outcomes.

## Methods

The evaluation used a before-after comparison design without control. This paper analyses data about the composition of VHSCs at both baseline and end-line, using data from all the 1186 VHSCs in the two districts. This information was collected from a VHSC office bearer at baseline, and from the available records at end-line. This paper also analyses the participation of VHSC members in its activities, comparing responses from three each randomly selected VHSC functionaries and non-functionaries from 150 each randomly selected VHSCs in two districts at baseline and end-line.

## Results

Most outcomes have shown significant improvements at the follow-up survey. For instance, there was an 84% increase in VHSC membership, from about 14000 members at baseline to about 26000 members at end line. The proportion of non-functionaries among the members increased from 54% to 64% (p<0.01). Similarly the representation of scheduled caste and scheduled tribe representatives among the non-functionary females has increased from 40% to 44% (p<0.01). The differences are more pronounced with regard to the VHSC activities. For instance, while only 3% of the VHSCs had reported having organized Jansamvada (platform to bring the providers face to face with the community) at baseline, this proportion has increased to 97% at end line. According to the non-functionaries interviewed at end line, 64% had reported that the VHSC had met at least once a month in the past one year, compared with 8% at baseline.

## Discussion

The intervention employed a set of resource persons (local men and women, trained in community participation in health) who mentored the VHSCs for two years, focusing largely on the structure and composition of VHSCs as per the guidelines, importance of community participation and monitoring, and the conduct of regular meetings. Activities like Jansamvadas and VHSC meetings where issues of health and quality of services at the village level are discussed increases the transparency and accountability of health systems and results in an increased joint responsibility of community and grassroot health functionaries to fill gaps in the current system. Grassroot community structures such as VHSC provides opportunity for issues of marginalised groups such as scheduled caste and scheduled tribe women to be represented and also bring them within the health service radar.

## Competing interests

None declared

## Funding statement

This study was funded by the Government of Karnataka.

